# Supplementary material for: Are carbon emissions trading and green financial instruments synergistic? -Comprehensive quantitative research based on content analysis
Source: PLoS One. 2024 Mar 7;19(3):e0298601. doi: 10.1371/journal.pone.0298601 (PMC10919601; doi:10.1371/journal.pone.0298601)
Supplement: S1 Data — (ZIP) [file pone.0298601.s001.zip › Data/地级市Z_F工作报告.zip!/╜¡╦╒/╦▐╟¿╩╨/└┤╫╘╣1⁄2╓┌║┼í╛┬φ┐╦╩2╛▌═°í┐_2009.docx]

各位代表：

现在，我代表市人民政府，向大会作工作报告，请予审议，并请市政协各位委员和其他列席人员提出意见。

2009年，是新世纪以来，我市经济社会发展经受挑战最多、经历困难最大的一年，也是我们团结和带领广大干部群众解放思想、砥砺奋进、攻坚克难，经济社会发展取得丰硕成果的一年。面对国际金融危机的扩散蔓延和国内外形势的复杂变化，在市委的坚强领导下，我们坚决贯彻中央和省一系列决策部署，在应对挑战中抢抓机遇，在创新思路中破解难题，在激烈竞争中加快发展，圆满完成了市三届人大二次会议确定的各项任务，提前一年实现了“十一五”规划的主要目标。

——综合实力跃上新的平台。预计实现地区生产总值超过810亿元、增长14%以上，人均GDP超过2500美元；财政总收入首次突破百亿元大关，达到130.2亿元，增长41.3%；一般预算收入在全国333个地级市排名中首次进入百强行列，达到63.1亿元，增长35.6%；90%左右的乡镇财政总收入超过千万元。

——产业结构发生积极变化。预计三次产业比例达到18.7：47.6：33.7，农业占比首次降至20%以下，二产、三产占比分别提高1个和0.7个百分点。传统产业支撑作用明显，新兴产业不断发展，高效农业、现代服务业也都有了突破性的进展。

——人民生活水平显著提高。预计城镇居民人均可支配收入12320元、农民人均纯收入6080元，均增长12.5%。各级财政用于民生的投入超过70亿元，增长30%。

　　过去的一年，我们主要抓了以下几方面工作：

一是抢抓机遇扩大有效投入，大力培育新的经济增长点。预计全年完成50万元以上固定资产投资745亿元，增长41.1%。扎实开展“亿元大项目推进年”活动，分别新引进、开工、竣工亿元以上工业项目289个、57个和22个，累计完成工业固定资产投资556.3亿元，增长40.9%。翔盛粘胶、德华纺织、金鑫轧钢等一批大项目相继投产，可成科技、晨风服饰、赐富薄膜等一批大项目进展顺利。不断深化南北挂钩合作，全面推进共建工业园区建设，新落户来自苏州的产业转移项目61个，到位资金35.3亿元。全面开展“千名干部帮千企”活动，及时兑现工业发展扶持奖励政策，支持企业加快发展。全年净增规模以上企业357户，总数达到2035户，产值超亿元企业达到110家，实现规模以上工业增加值198亿元，增长20.2%。洋河股份成功上市，双沟酒业销售收入突破25亿元，“箭鹿”、“绿陵”被认定为中国驰名商标。扎实推进农业产业化经营，加强龙头企业建设，促进高效农业发展。广东温氏、北京汇源等知名企业相继落户，146家市级以上龙头企业累计实现销售收入76.3亿元，增长10.5%。粮食生产连续第六年保持增产，新增高效农业36.3万亩、高效渔业11.3万亩。认真落实汽车下乡、家电以旧换新等刺激消费政策，城乡市场繁荣活跃，汽车类消费实现翻番，社会消费品零售总额达到234.4亿元，增长19.1%。获批省级软件和信息服务产业园3家、省级国际服务外包示范区2家，软件和服务外包产业实现主营业务收入5亿元，增长4倍。完成进出口总额6.23亿美元，其中出口5.35亿美元，分别增长31%和24.3%。

二是统筹推进城乡建设，不断增强区域发展的载体支撑。立足于优化城市功能、改善城市环境、提升城市品位，全面推进配套服务设施建设。市区的金柏年财富广场、四季青服装批发市场以及沭阳县的大润发、泗洪县的“第一街”、泗阳县的苏果等大型商业设施相继投入运营，海关国检大楼、湖滨新城商务大厦完成主体工程，宝龙城市广场正加快建设。中心城市水系沟通、市区第二水厂等重点水务工程进展顺利，一批生态环保基础设施投入使用，全市污水总处理能力达到25.5万吨/日，完成省下达的年度节能减排任务，秸秆禁烧再次实现国家卫星遥感监测零火点记录。荣膺“中国优秀旅游城市”称号，再获“中国城市管理进步奖”殊荣，顺利通过“国家园林城市”现场专家评审。在加快中心城市和县城建设的同时，制定出台鼓励农民进城（中心镇）购房和到集中居住区建房居住的政策，合理引导农民住房需求，全市共启动建设农民集中居住区638个，累计建房5万余户，城市化率提高到37.8%。省道245、249及市区运河一号桥、黄河三号桥等重点交通工程顺利推进，期盼已久的宿宿淮铁路、宿新高速启动建设，宿迁融入长三角、对接沿海开发迈出了实质性步伐。

三是着力深化改革创新，有效破解发展瓶颈制约。加大金融领域改革创新力度，引导各金融机构扩大信贷投放。引进苏州五家农商行到宿设立分支机构，重组市国丰、城投、交投、水投、国投五大投融资平台，新成立5家注册资本亿元以上担保机构，7家小额贷款公司开业运营，全市金融机构各项贷款余额比年初净增142亿元，增长43.8%，比全省平均水平高出8.9个百分点。积极推进城乡建设用地增减挂钩，实施“万顷良田”建设工程，深入开展土地“三清”工作，促进城乡土地资源的合理配置和高效利用，全年新增建设用地储备面积2.46万亩，盘活存量土地8000亩。继续深化行政审批制度改革，削减行政审批事项，压缩审批办理时限，市级行政权力实现网上公开透明运行。创新中心城市管理体制和运行机制，赋予宿豫区、宿城区与各县同等的经济社会管理权限，把宿迁经济开发区、市湖滨新城作为相对独立的经济社会发展主体，放手放权支持四个城区加快发展。

四是健全完善社会保障体系，尽最大努力保障和改善民生。实施更加积极的就业政策，突出以创业促就业、增加投入保就业、政府购岗助就业，全年新发展私营企业6709家、个体工商户4.86万户；城镇新增就业3.2万人，登记失业率3.1%；新转移农村劳动力12万人，返乡农民工全部实现就业。覆盖城乡的社会保障制度框架基本形成，保障范围不断扩大，标准逐步提高。全市“五险”扩面合计新增17.5万人，3.8万农村居民参加新型社会养老保险试点，城镇居民医疗保险参保率、新型农村合作医疗参合率均达98%以上。企业退休人员人均养老金提高到每月1028元，失业保险金最低发放标准提高到每人每月371元，职工大病医疗救助、城镇居民医疗保险最高支付限额分别提高到20万元和10万元，城乡低保标准提高到每人每月210元和130元，农村五保集中和分散供养标准提高到每人每年2700元和2100元、集中供养率达61%。新建经济适用房52.6万平方米，新筹集廉租住房874套，人均月收入低于450元、住房建筑面积低于16平方米的低收入住房困难家庭全部纳入住房保障范围。又有54.3万农村人口喝上干净卫生的自来水，26万农村贫困人口实现脱贫。全力支援四川地震灾区灾后重建，对口援建的绵远小学、医院等6个项目在全省率先全面竣工并投入使用。

五是积极发展各项社会事业，切实维护和谐稳定良好局面。及时兑现义务教育教师绩效工资，大力扶持民办教育发展，城乡义务教育全部免除学杂费，春秋两季累计发放免费教科书128万套。义务教育入学率保持100%，初中毕业生升学率连续五年保持苏北第一，高考本科达线突破万人大关。农村留守少年儿童食宿条件改善和农村合格幼儿园建设“两项工程”惠及留守儿童10万余名、幼儿8万余名。10所职业学校被评为省三星级以上职业学校，市技工学校通过国家高级技工学校验收，湖滨新城职教园区在校生超过3万人，全市普职比达到4.3:5.7。认真贯彻中央和省深化医药卫生体制改革精神，加强公共卫生服务体系建设，健全市县乡村四级医疗卫生服务体系，推行基本药物制度，市传染病防治中心投入运行，手足口病、甲型流感得到有效防控。新建乡镇卫生与计生服务中心99家，计划生育率达到95%以上。与南农大、南工大、南信大等多所高校建立产学研合作关系。大型音舞诗剧《虞美人》在宁首演，与电影《彭雪枫纵横江淮》、电视剧《美丽的中国结》同获省“五个一工程”奖。在苏北率先全面完成乡镇文化站建设，农家书屋建设实现全覆盖，有线电视新发展农村用户13.9万户，市博物馆、名人馆免费对外开放。扎实推进平安宿迁、法治宿迁建设，建立健全应急管理机制，各类社会矛盾及时化解，安全生产形势总体良好，食品药品市场平稳有序，社会保持和谐稳定。沭阳县、泗阳县分别被评为“全国文明县城”和“全国平安建设先进县”。反腐倡廉建设不断加强，国防动员、人民防空、双拥共建工作取得新的成绩，统计、审计、档案、物价、气象、工会、妇女、青少年、工商联、地方志、民族宗教、外事侨务、对台事务、无线电管理等事业也都取得新的进步，为全市经济社会发展作出了新的贡献。

各位代表，回首过去一年，全市经济社会能够在复杂严峻形势下保持快速发展的好势头，逆势跃上新平台，确实来之不易，十分难能可贵。这些成绩的取得，是市委正确领导和市人大、市政协有效监督、全力支持的结果，集中了方方面面的智慧和力量，凝聚了全市上下的心血和汗水。创业的路上充满了艰辛、挑战与考验，也留下了我们共克时艰、共谋发展、共创和谐的奋斗足迹。我们不仅全面完成了促增长、促发展的预期目标，而且在应对危机中提高了驾驭复杂局面的实际能力，在改革创新中积累了破解难题的重要经验，为做好今年乃至“十二五”时期的工作奠定了良好基础。在这里，我代表市政府，向在各条战线上付出辛勤劳动的全市人民，向监督支持我们工作的人大代表、政协委员以及各民主党派、工商联、群众团体和离退休老同志，向为宿迁改革发展稳定作出积极贡献的中央和省驻宿单位以及人民解放军、武警官兵和人民警察，向直接参与宿迁建设的海内外客商、省级机关和苏州市广大援宿干部，以及所有关心支持宿迁发展的社会各界人士，表示衷心的感谢并致以崇高的敬意！

各位代表，在充分看到成绩的同时，我们也清醒认识到工作中存在的矛盾和问题：相对于经济总量的快速膨大，产业质态还需进一步提高，新兴产业发展比较滞后，经济增长的外向拉动力仍然不足；相对于工业化水平的快速提升，城市化进程还需进一步提速，全市城市化率明显低于全省平均水平，中心城市和部分县城的人气商气需要加快集聚；相对于财政收入的快速增长，富民步伐还需进一步加快，城乡居民收入增幅总体上低于财政收入的增幅；相对于经济社会的快速发展，节能减排工作还需进一步加强，全面完成“十一五”节能减排任务必须付出更大努力。同时，少数政府部门的服务意识有待进一步增强，办事效率有待进一步提高，形式主义、官僚主义和消极腐败现象在少数人身上仍不同程度地存在。对于这些问题，我们一定给予高度重视，采取有效措施，切实加以解决。

2010年是全面完成“十一五”规划、超前谋划“十二五”发展的关键一年。今年政府工作总的指导思想是：坚持以邓小平理论、“三个代表”重要思想和科学发展观为指导，认真贯彻中央和省经济工作会议精神，按照市委三届五次全体（扩大）会议的部署，着力构建现代产业体系，加快经济转型升级步伐；加速推进城市化进程，增强统筹城乡发展能力；全面深化改革开放，形成更加有利于科学发展的体制机制；切实保障和改善民生，促进社会和谐稳定；全力冲刺经济总量、固定资产投资双超“千亿”的新高峰，坚定不移地推动经济增长继续走在全省前列。

经济社会发展的主要预期目标是：地区生产总值增长13.5%，财政总收入增长30%以上，一般预算收入增长25%以上；50万元以上固定资产投资增长35%以上；社会消费品零售总额增长18%，进出口总额、实际到账外资均增长15%以上；研发投入占地区生产总值比重达到0.6%以上，高新技术产业产值占规模以上工业比重达到6%；城镇居民人均可支配收入增长12%以上，农民人均纯收入增长11%以上；万元GDP能耗下降5%以上，COD、二氧化硫排放总量在2005年的基础上分别削减8.3%和2.8%。

这些指标的安排，既是确保经济增长继续走在全省前列的客观需要，更是宿迁实现更大突破的根本要求；既全面分析了宿迁保持较快增长的现实可能性，也统筹考虑了各方面的不利因素和有利条件。就目前来看，今年经济社会发展环境尽管仍然存在一些不确定、不稳定因素，但总体上将好于去年。国际金融市场渐趋稳定，世界经济有望实现恢复性增长。中央宏观经济政策的基本取向没有改变，积极的财政政策和适度宽松的货币政策将继续实施。宿迁已经整体纳入长三角发展一体化规划，与沿海开发正紧密对接，在国家宏观发展战略中的地位明显提升；财政总收入超百亿，一批重大项目正加快建设，实现更好更快发展的基础和能力有了新的增强。我们只要牢牢把握发展机遇，充分发挥比较优势，坚持不懈，埋头苦干，就一定能够圆满完成全年目标任务。

在今年工作中，将注意把握好以下五个方面：一是更加注重在狠抓投入中培育新的增长点。切实利用好积极财政政策和适度宽松货币政策的机遇，以项目推进为主线，以有效投入为抓手，积极稳妥地加大政府投入力度，激发社会投资活力，增加信贷投放规模，着力培育更多的新经济增长点，真正以高投入拉动高增长，以大项目推动大发展。二是更加注重在转型升级中构筑产业优势。以提高增长质量为目标，切实处理好速度和效益的关系，在尽可能快地扩张经济总量的同时，以前瞻性的眼光和思路，主动超前实施科技自主创新，不失时机推进经济转型升级，加快建立以传统产业为主体、新兴产业为主导的特色产业体系。三是更加注重在统筹兼顾中加快城市化进程。在扭住新型工业化这个核心不动摇的同时，下更大力气推进城市化，特别是集主要精力建设好中心城市和县城，以产业的集聚发展增强城市承载能力，通过城市的功能内涵提升为产业发展提供配套服务，着力构建以中心城市为核心、中小城市和重点城镇为纽带、农民集中居住区为基础的城镇化体系，实现工业化、城市化的相互促进和良性互动。四是更加注重在改革开放中激发内生动力。深入推进新一轮思想解放，推动重点领域和关键环节的改革取得更大突破，打造更具吸引力的投资环境，以更加有效的制度安排助推科学发展、跨越发展。矢志不移地走开放兴市之路，大力实施“东向战略”，更加主动地融入长三角一体化、对接沿海大开发，全面拓展对外开放的广度和深度，提高对外开放的质量和水平，推动外向型经济再上新台阶。五是更加注重在加快发展中全力改善民生。把保障和改善民生作为最重要的政策取向和政绩追求，依靠发展经济为改善民生创造条件，通过民生改善为经济发展提供动力。坚持既尽力而为又量力而行，更加积极地推动公共资源配置向民生领域倾斜，不断完善与经济发展水平相适应的社会保障体系，让发展的成果更多地惠及于民，切实增强人民群众的幸福感和满意度。

全力推动经济社会跨越发展

　　围绕保增长、扩内需、调结构、促转型、惠民生的总体要求，突出重点，统筹兼顾，着力做好六个方面工作。

　　一、坚定不移引大培强，打造工业主导产业新支撑

　　突出以大项目带动大产业。确保食品饮料、纺织服装、林木加工、机械电子四大百亿级产业，年内分别实现销售收入200亿元、150亿元、150亿元和100亿元。始终把招商引资作为第一抓手，组织精干力量组建6个专业招商局，大力开展“亿元大项目攻坚年”活动，采取协作组招商、产业链招商、专业招商、以商引商等方式，迅速掀起招商引资新热潮。瞄准国内外大企业、大集团，重点加强与央企、省属大企业对接，着力引进一批产业关联度大、技术含量高、辐射带动力强的龙头型项目。全年确保新引进、开工、竣工亿元以上项目300个、150个和100个，每个县（区）引进投资15亿元以上的大项目不少于2个，全市工业固定资产投资增长50%以上。推动在手重大项目加快建设，力争可成科技一季度首条生产线投产，天威节能、晨风服饰、宝迪食品上半年投产，翔盛粘胶二期、德力化纤、明宇电气、苏钢鑫益机械下半年投产，林浆纸一体化项目力争年内开工。

　　大力培育重点骨干企业。研究出台更具针对性和含金量的“短平快”政策，加大对本土企业和已落地生根外来企业的扶持力度，鼓励企业通过技术改造、购并联合做大做强。选取20家具备上市潜力的企业进行重点培育，引导企业通过发行企业债券等方式直接融资。重点支持40个省级、50个市级新增长点建设，帮助中小企业成长壮大，全年确保净增年销售收入超亿元企业20家、规模以上企业350户，规模以上工业增加值增长20%以上。强化经济运行调节，完善落实扶持企业发展的政策措施，做好煤电油运调度和生产要素保障，促进企业效益持续好转。今年将继续增加新型工业化产业引导资金，全市各级财政直接安排的资金总额不低于1.5亿元。

　　全面提升园区发展水平。强化基础功能配套，认真研究市内尤其是市区各开发区资源整合和布局优化问题，促进功能叠加、资源共享和优势再造，增强大项目落户承载力。严格坚持投入产出标准，提升集约发展水平，推动开发区全面升档进位，今年各省级开发区在全省综合排名至少上升3个位次。以南北共建园区为主要载体，积极创建“江苏省产业转移示范市”，把宿迁打造成为苏南等发达地区产业转移的最佳集聚区，每个共建园区今年分别引进5亿元以上项目1个、亿元以上项目5个。大力支持洋河、双沟两大特色园区建设，增强酒类产业竞争力，全力打造“酒都宿迁”品牌。促进重点中心镇工业集中区健康快速发展，每个县（区）重点培育3—5个乡镇工业集中区。

增强外向型经济拉动作用。坚持抓外资、促外贸、带外经，积极开展“外资突破年”活动，重点加大对台招商力度，努力争取举办“台湾江苏周”专项经贸洽谈活动，全力打造台资企业新集聚区。年内确保实际到账外资1.8亿美元，每个县（区）引进投资6000万美元以上的外资项目不少于1个。积极拓展外贸市场，鼓励企业参加各类展会，对出口实绩明显、发展潜力较大的企业进行重点扶持，全年实现进出口总额7亿美元。大力实施“走出去”战略，全方位参与国际经济交流与合作，确保全年实现对外经济合作营业额3500万美元以上。强化外向型经济载体建设，海关国检综合楼确保年内投入使用，海关机构正式开关运作，国检机构力争尽快获批；依托运河宿迁中心港枢纽工程，积极论证规划和启动建设宿迁口岸。

　　二、扎实推进农业产业化经营，增创高效规模农业新优势

　　强化龙头企业建设，推进高效农业规模化。全力招引国内外知名的“农”字号大企业落户，每个县（区）年内引进不少于1个投资亿元以上的龙头企业项目，全年农业招商项目到位资金40亿元。开展“龙头企业提升年”活动，加快中加奶牛、江苏雨润、南京卫岗等项目建设。充分发挥财政资金引导作用，按照“整合打包、集中扶持、整体推进、连片开发”的要求，着重向重点龙头企业、产业核心基地以及合作组织、种养大户倾斜，提高现代高效农业区域化、规模化、专业化发展水平。全年确保新增高效农业40万亩、高效渔业15万亩，新发展设施农业20万亩，每个县（区）至少建成1个万亩连片的省级现代农业产业园。推进土地流转有形市场规范化建设，大力发展农民合作经济组织，促进农业适度规模经营，年内农民合作经济组织新增入社农户10万户以上，新发展适度规模经营45万亩。

　　发挥科技引领作用，提高农业的质量和效益。大力实施农业新品种、新技术、新模式“三新”工程，支持市农科院发挥棉花品种创新等科研优势，加快“运河湾科研基地”建设，打造全省一流的现代农业科技示范园区和国内领先的国家级棉花育种科研基地；以荷兰绿港、瑞克斯旺等项目为主导，加大优质种苗研发推广力度，把宿迁培育成为重要的种苗“三新”示范基地。年内每个县（区）都建成一个集精品生产、科技示范、教育培训于一体的农业科技示范园。强化农产品质量安全建设和品牌创建，对获得省级以上质量或品牌认证的农产品生产主体给予一定奖励，年内每个县（区）确保创建省级名牌农产品2个以上。积极发展循环农业，结合沼气工程建设，加强种养产业之间衔接，实现污染减排、资源循环，促进农业经济持续稳定发展。年内新增农村户用沼气池3000处以上、规模畜禽场沼气治理工程20处以上。切实抓好重大动物疫病防控，保障畜牧业健康稳定发展。

　　健全流通服务体系，促进农产品产销衔接。加强农产品交易市场建设，每个县（区）年内都建成1个交易面积3万平方米以上、年交易额1亿元以上的特色农产品交易市场，并在年内投入运行。推进农产品网络销售平台建设，强化信息服务，培育无形市场。积极借助江苏农业国际合作洽谈会、江苏名特优农产品（上海）交易会、中国国际农产品交易会等会展平台，促进农产品生产基地、加工企业与市内外批发市场、连锁超市的合作对接，不断拓展农产品销售渠道。

　　三、积极推动现代服务业发展，扩大消费需求新空间

　　坚持外引与内培并举、生产性与生活性并重，推动服务业发展提速、比重提高、结构提升。全年确保实现服务业增加值320亿元，增长15%以上，占GDP比重提高1.5个百分点。大力发展软件和服务外包产业，组织实施好宿迁软件园二期、三期工程，宿迁服务外包产业园大型呼叫中心、电子商务中心、数据备份中心和软件孵化器的建设，着力推进京东商城（宿迁）信息科技园、经纬服务外包产业园呼叫中心等重点项目，全年新增软件和服务外包企业100家，主营业务收入突破10亿元。高度重视现代物流业发展，加快开发区物流园、粮食物流中心二期建设，培育2家以上具有区域辐射功能的物流基地、5家以上第三方物流企业，鼓励条件成熟的农村地区建设客货兼顾的道路运输场站。着重加强洪泽湖湿地公园、苏北花卉生态旅游园等重点旅游项目建设，扎实推进骆马湖水上旅游开发，努力打造具有地方特色和核心竞争力的旅游品牌，推动旅游业有更大发展。继续采取强有力的措施，增加普通优质商品房的有效供给，规范房地产市场秩序，有效防止商品房价格过快增长，促进房地产业健康发展，全年新增房地产开发面积650万平方米。加快演艺娱乐、动漫制作、文化博览、影视广播等文化产业发展，尽快实现新的突破。

　　不断拓展消费需求空间，在千方百计增加城乡居民收入，增强居民消费能力的基础上，认真落实扩内需政策，简化汽车摩托车、家电下乡和农机购置补贴程序和操作流程，做好家电以旧换新工作，巩固发展城乡消费持续增长的好势头。进一步健全城乡商贸流通体系，实施“万村千乡市场工程”，重点刺激农村消费，优化消费环境，发展新型消费业态，培育汽车、旅游、住房等消费热点，促进市场持续繁荣。大力发展纺织品、服装、家具、建材等具有地方特色的专业市场，加快餐饮酒吧、休闲会所、教育培训、医疗保健等服务场所建设，引导多样化消费需求。

　　四、全面提升基础设施配套水平，构建城乡一体化发展新格局

　　完善区域交通网络。加快构筑公、铁、水、空一体化的现代综合交通运输体系，全年完成交通基础设施总投入25亿元。在公路建设上，开工建设沭阳至连云港的省道344宿迁段，做好省道326建设前期工作，着力打造宿迁向东连接连云港机场和港口的无收费快速通道。加快省道245、249宿迁段建设，确保年内竣工。开工建设国道205、省道330、325宿迁段，按计划推进宿新高速建设。年内完成农村公路通车里程299公里。在铁路建设上，全力配合宿宿淮铁路建设，力争明年底建成通车；完成徐宿淮铁路立项审批工作，争取早日开工建设，把宿迁建设成为华东铁路网中的重要节点城市。在水运建设上，加快建设宿迁运河中心港物流枢纽，开通运河集装箱运输。规划建设宿迁至连云港港口的水上快速通道，构筑通江达海的大港航格局。积极争取在连云港港口设立宿迁码头区，在宿迁设立对接连云港港口的物流服务及货代区，把宿迁打造成直接承接国际海运的“无码头港口”。在航空建设上，加强和徐州、连云港机场共建共享，年内确保徐州观音机场宿迁城市候机楼投入使用，并开通旅客班车，实现“一站式”便捷化服务，最大限度方便旅客出行、货物运输。

　　丰富中心城市功能和内涵。在积极推进第三轮城市总规修编、强化规划执行的基础上，着重围绕人气商气集聚，推进总投资269亿元的中心城市六个方面74项重点工程建设，年内确保完成投资110亿元以上。一是滨水景观营造工程。举全市之力建设湖滨新城，确保省第七届园博会园博园完成总工程量80%以上。千方百计加快中心城市水系沟通工程建设，今年确保全面开工，力争五年任务三年完成。二是危旧片区改造工程。全年市区新建拆迁安置房不低于150万平方米，其中“幸福公社”一期415亩危旧片区改造工程确保一季度启动，年内投入不低于8亿元，完成住宅及配套工程20万平方米以上。三是城市客厅靓装工程。全面推进宝龙城市广场、金鹰天地广场暨国际购物中心、金田国际广场、中豪国际广场建设，加快形成城市中心区黄河四号桥至项王桥段城市地标群。四是历史街区修复工程。精心组织宿迁“1897”、粮食博物馆、水利博物馆、酒文化博物馆等工程建设，积极申报国家级和省级文保单位，加快推进历史文化名城创建工作。五是迎宾门户拓展工程。重点实施好开发区大道景观优化、环城西路道路优化及景观绿化提升等项目建设。六是基础功能提升工程。全面加快市区水、气、路、管网及文体场馆等基础设施配套，按计划推进总投资23.2亿元的重点电网工程建设，完成市区第二水厂净水厂及配套管网建设，强化城市基础支撑。沭阳、泗阳、泗洪三个县城也要超前谋划和推进一批重点基础设施项目，不断提升综合承载能力。

　　提高镇村建设发展水平。按照城乡一体化和“集约、集聚、集中”的发展理念，全面启动新一轮小城镇规划修编，完成所有确定的农村集中居住区详细规划编制，构建科学合理的市域城镇体系和村镇规划体系。把省、市重点中心镇作为小城市打造，在产业布点及社会事业资源配置上予以倾斜，吸引周边地区农民就业落户。积极探索农民原居住用地的退出补偿机制，通过集中投入、连续投入，加快集中居住区服务设施配套，促进建新与拆旧衔接推进，新房入住与老宅置换同步落实，确保年内具备入住条件的集中居住区入住率达60%以上、老宅基置换率达80%，每个集中居住区达到500户、人口在2000人以上。加强农业资源开发，大力改造中低产田，积极推广新型农机具，完善水利设施配套，提高农业综合生产能力。

　　加强生态建设和环境保护。深入开展低碳示范城市、国家园林城市、国家级环保模范城市以及“江苏人居环境城市管理范例奖”创建活动。认真落实河（湖）长制度，深入实施节能减排攻坚工程，确保“十一五”节能减排任务不折不扣完成。沭阳县城南污水处理厂、泗洪县城北及双沟镇污水处理厂、宿城区洋河污水处理厂二期和新区污水西接工程、宿迁经济开发区配套污水管网工程，以及市湖滨新城生活污水处理厂等重点工程确保上半年完工，全年新增日处理污水能力16.75万吨，新建排污管网145公里，市区污水收集处理率达到85%以上，各县城区达到75%以上。大力发展循环经济，推行清洁生产，淘汰落后高耗能设备，提升建筑节能水平。持续开展“杨树产业年”活动，高标准推进城市绿化建设，强化农村环境综合整治，大力开展环境优美乡镇、村庄创建活动，不断优化、美化城乡生活和发展环境。

　　五、继续深化重点领域和关键环节的改革创新，增添科学发展新动力

　　推进财政金融改革与发展。强化财政收入监管，深化预算管理和非税收入管理改革，开展财政资金绩效评估，发挥财政资金的酵母和杠杆作用，引导更多的社会投资投向重点领域、重点项目和重点工程，提高财政资金使用效益。认真做好金融改革试点，加大金融招商力度，年内确保引进金融机构2家，组建村镇银行2家，成立农村小额贷款公司8家，2家以上农村合作银行改制为农村商业银行，力争交通银行、南京银行、华夏银行等股份制商业银行来宿设立分支机构。积极争取扩大授信额度，增加贷款规模，全年新增贷款200亿元以上，其中工业贷款60亿元以上。继续加强投融资平台建设，切实增强融资功能和投资经营能力，到年底，市级五大投融资平台总资产不低于150亿元，直接融资30亿元以上。探索开办“金融超市”，在银行与小企业、个体工商户和自主创业者之间搭建融资平台。定期组织银政、银企、银保对接，全年组织银企洽谈会3次以上，为企业融资突破200亿元。激励和引导中小企业认缴互助基金，年内基金规模达到5000万元，互助融通资金额度达5亿元。全面推进政策性农业保险，扎实做好第二批农业险种试点扩面工作。

　　促进土地资源节约集约利用。实施差别化供地政策，将用地计划向技术含量高、产出效益好、环境污染少的项目倾斜，重点保障工业大项目、城市基础设施建设、民生保障工程等用地需求，切实提高用地绩效，促进土地节约集约利用。加快“万顷良田”工程建设，年内完成建设总量70%以上；积极争取城乡挂钩置换周转指标，全年新增2.5万亩以上。推进开发区土地清理整顿，继续做好“三清”工作，加大对闲置、囤积土地的整治力度。进一步完善基本农田保护考核评价体系，抓好土地整理项目建设督查、检查和验收，确保耕地占补平衡有余。加大土地收购储备经营力度，充分发挥储备土地融资作用，为重大基础设施、旧城改造筹措建设资金。积极开展农村集体建设用地收储和开发，促进城乡建设用地资源合理配置。

　　加快自主创新体系建设。着力构建以企业为主体的自主创新体系，立足实际走引进消化吸收再创新之路，不断增强区域自主创新能力。积极吸引国内大企业大集团来宿创办、支持本地企业与高校院所联办科研机构，推动规模以上企业建立研发机构、开展产学研合作，全年实施产学研合作项目200个以上。加强科技公共基础设施建设，确保年内国家白酒产品质量监督检验中心基本建成，江苏省（洋河）生物酿酒技术研究院启动建设，全年实施省、市重点技术创新项目25个。围绕主导产业和新兴产业发展，大力实施“百名创业创新领军人才集聚计划”，确保年产值5000万元以上企业全部拥有长期合作的外协专家。

　　六、着力加强以民生为重点的社会建设，努力开创和谐稳定新局面

　　优化服务推动创业就业。全面落实以创业促就业政策，严格按规定加大创业小额担保贷款支持力度，放大以创业带动就业的倍增效应，年内确保每个县（区）都设立总额不少于500万元的创业小额贷款担保基金，为各类有创业愿望的群体提供有效、便捷的服务，真正使想创业的有机会、敢创业的得支持、创成业的受鼓励，全年新增个体工商户3万户、私营企业3000家。继续组织实施特别培训计划，全年完成农村劳动力培训4万人、再就业培训2万人。加大就业再就业资金投入，全力做好重点企业用工服务，继续通过政府购岗等方式做好就业困难群体就业援助工作，全年新增城镇就业2.2万人，失业人员再就业8000人，城镇登记失业率控制在4%以内，动态消除城镇零就业、农村零转移家庭。市、县（区）安排就业再就业资金比去年增加10%以上。加快建立城乡一体的公共就业服务体系，把公共就业服务网络延伸到村，年内30%以上的行政村设立劳动保障工作站。扩大失业保险基金支付范围，增加用于促进就业的支出比例，充分发挥失业保险的“保生活、促就业、防失业”功能。

　　尽力提高社会保障水平。全面实施新型农村社会养老保险，足额安排补助资金，高标准推进新农保制度全覆盖，确保参保率达到95%以上，农村老年居民基础养老金年内全部发放到位。落实国家调整企业退休人员养老待遇政策，确保养老金按时足额发放。做好三产服务业、商贸流通业、新型经济组织、社会中介机构以及灵活就业群体社会保险扩面工作，全年“五险”扩面合计新增13万人以上。建立城镇职工基本养老保险关系转移接续平台，确保参保人员养老保险关系无障碍转移接续。不断提高城镇居民医疗保险待遇水平，把城镇职工、居民报销比例分别提高到80%和60%，做好城市规划区范围内城镇居民参加医保工作，确保参保率达98%、参保人员续保率达90%以上；提高新型农村合作医疗补偿比例，将筹资标准提高到150元，其中财政补助120元，确保参合率稳定在98%以上；建立医保异地就医联网结算系统，年底前在全市范围内实现城镇职工、居民医保参保人员就医购药“一卡通”。认真落实被征地农民社会保障政策，实现新征地农民“即征即保”。按自然增长机制要求提高城乡低保和农村五保供养标准。增加保障性住房供给，全年新增廉租住房668套，新开工经济适用住房5600套，确保人均月收入低于600元、住房建筑面积低于18平方米的低收入住房困难家庭全部纳入住房保障。全面加强社会救助，加快残疾人事业发展，切实保障特殊困难群体基本生活。继续实施饮水安全工程，年内新解决50万农村人口饮水安全问题。深入实施“脱贫攻坚”工程，确保年内再有26万以上农村贫困人口实现脱贫。

　　协调发展各项社会事业。加快教育现代化创建步伐，力争泗阳、宿城率先通过省级验收；做精做优基础教育，完善农村教育保障机制，保证城乡小学和初中的生均公用经费分别达到370元、610元，义务教育阶段普及率、巩固率保持100%；抓好中小学校舍安全工程建设，全年完成300万平方米改造任务；宿迁学院年内申本成功；提升职业教育办学水平，推进职业教育集团化办学，全市普职比达到4：6；落实对民办学校以奖代投政策，促进民办教育可持续发展。加快建立基本药物制度，按照人均不低于15元的标准安排基本公共卫生经费，不断扩大基本公共卫生服务覆盖面；市人民医院病房大楼年内建成，完成104个乡镇卫生院升级改造和村卫生室改建任务；充分发挥市传染病防治中心的作用，实现市区传染病人集中收治；成立市、县医疗卫生事业投资管理中心，统一负责各级政府对医疗卫生机构投入资金的使用和管理，实现政府资本投入与社会资本投入的有效对接。加快计划生育服务体系规范化建设，年内“世代服务”中心全部投入使用，持续稳定低生育水平。扎实开展全国第六次人口普查。积极繁荣文化事业，大力培育文化企业，加快“文化宿迁”建设。认真贯彻落实全民健身条例，加强城乡公共体育设施建设，扎实做好十七届省运会参赛工作。

　　构建维护稳定的长效机制。深入开展基层信访工作达标升级活动，抓好乡（镇）信访基础性工作，努力减少基层信访量。继续坚持领导干部定期接访、下访制度，健全领导包案机制，积极化解信访积案。大力推进劳动保障监察“两网化”和劳动争议仲裁“实体化”建设，严格执行农民工工资公示、保证金、专用账户制度，推行工程建设领域“黑名单”制度，确保不发生农民工工资拖欠问题。深入推进平安宿迁、法治宿迁建设，强化社会治安综合治理，加强法律服务和援助工作，着力构建城乡一体的社会治安“大防控”格局。强化食品药品安全监管，规范食品药品市场秩序，确保群众饮食用药安全。加强价格监测预警体系建设，整顿规范市场价格，净化消费环境。全面落实安全生产责任制，加大对道路交通、危化品、建筑施工等重点行业的整治力度，强化事故隐患排查治理，坚决遏制重特大安全事故的发生。高度重视应急管理工作，建立统一指挥、反应灵敏、协调有序、运转高效的应急管理机制，不断提高预防和处置突发公共事件能力。加强国防动员和民兵预备役建设，推动双拥共建。

各位代表，做好“十二五”规划编制工作，是今年的一项重要任务。我们将在提前一年实现“十一五”规划主要目标的基础上，充分发扬民主，凝聚各方共识，集中全民智慧，聘请专家参与，以严谨务实的态度，努力使规划更加科学完善，更加符合实际，更加具有前瞻性、战略性和导向性，真正编制出引领宿迁未来发展，惠及宿迁人民群众的好规划。

进一步提高政府管理和服务水平

　　新一轮跨越发展的宏伟目标，对政府工作提出了新的更高要求。我们将按照“为民、务实、廉洁、高效”的施政理念，坚持服务发展、服务群众，勤勉尽责、敬业奉献，开拓创新、奋发有为，不断开创政府工作新局面。

在政府职能转变上下更大功夫。根据省统一部署和要求，实施市、县（区）政府机构改革，理顺部门职责关系。认真落实向宿豫区、宿城区及宿迁经济开发区、市湖滨新城下放职权的有关文件，督促市直各相关部门加强业务指导，真正把权力下放到位。进一步完善行政审批“三集中、三到位”制度改革，特别是继续清理和规范涉企收费，凡是外地不收的，宿迁坚决不收；标准有弹性的，坚决按下限收取。坚持群众利益高于一切，下更大力气统筹解决好群众最关心、最直接、最现实的利益问题，今年将重点办好七个方面66项民生实事项目，全市各级财政用于民生的投入确保比去年增长30%以上。

　　在工作效能提升上下更大功夫。进一步修订完善政府工作规则，着重在强化领导之间分工合作、部门之间配合协作等方面，作出更加明确的规定，从制度上防止各自为政、推诿扯皮。大力推行“一线工作法”、“工作成果倒逼法”，真正把一件件实事、一个个项目、一项项工程抓紧抓实、抓出成效，确保政府承诺过的事情、定下来的工作坚决落实到位。坚持寓管理于服务之中，新建并运行好便民服务中心，办好管好“12345”市长热线，市级行政权力确保全面上网公开透明运行，各县（区）参照市级标准在年底前上网运行，最大限度地提高办事效率。

在科学民主决策上下更大功夫。自觉接受人大的法律监督和政协的民主监督，健全人大代表建议、政协委员提案的办理机制，强化办理责任，增强办理实效，在确保100%答复率的同时，着力提高满意率和办结率。经常深入基层、深入群众调查研究，问政于民、问需于民、问计于民，从基层和群众中汲取更多的智慧和更大的力量。坚持重大事项集体决策，健全民主集中、专家咨询、社会公示与听证等制度。从今年起，我们将以市政府的名义，聘请有关行业的顶尖专家，组建“外协专家顾问团”，为各主导产业发展提供智力支持。

　　在廉洁从政建设上下更大功夫。认真落实党风廉政建设责任制，深化教育、制度、监督并重的惩治与预防腐败体系建设。进一步规范公共资源交易行为，完善土地出让、政府采购、工程建设招投标等制度，下决心整顿和规范拆迁市场，坚决堵住一些重要部门、热点岗位、关键环节权力运行的监管漏洞。强化领导干部任中经济责任审计，加大对重点领域、重大工程、重要环节的审计和监察力度，严厉查处各类违法违纪行为，努力从源头上预防腐败。深入开展政风、行风评议活动和纠风治乱工作，对群众反映强烈的问题实行挂牌督办，责令限期整改。大兴艰苦奋斗、厉行节约之风，严控行政开支，降低公务成本，将更多财力用在刀刃上。通过努力，切实把我们的政府建设成为思想解放的政府，团结高效的政府，清正廉洁的政府，为民办实事的政府。

　　各位代表，新的目标催人奋进，新的任务光荣艰巨。让我们在市委的坚强领导下，万众一心，开拓奋进，苦干实干，合力攻坚，为全面完成今年各项任务而努力奋斗！
